# Automatic Recognition of Dendritic Solidification Structures: DenMap

**DOI:** 10.3390/jimaging6040019

**Published:** 2020-04-03

**Authors:** Bogdan Nenchev, Joel Strickland, Karl Tassenberg, Samuel Perry, Simon Gill, Hongbiao Dong

**Affiliations:** Department of Engineering, University of Leicester, Leicester LE1 7RH, UK; bn55@leicester.ac.uk (B.N.); jcjs2@leicester.ac.uk (J.S.); kt199@leicester.ac.uk (K.T.); sjp78@leicester.ac.uk (S.P.); spg3@leicester.ac.uk (S.G.)

**Keywords:** pattern recognition, image analysis, superalloy, 2-D quantitative analysis, directional solidification

## Abstract

Dendrites are the predominant solidification structures in directionally solidified alloys and control the maximum length scale for segregation. The conventional industrial method for identification of dendrite cores and primary dendrite spacing is performed by time-consuming laborious manual measurement. In this work we developed a novel DenMap image processing and pattern recognition algorithm to identify dendritic cores. Systematic row scan with a specially selected template image over an image of interest is applied via a normalised cross-correlation algorithm. The DenMap algorithm locates the exact dendritic core position with a 98% accuracy for a batch of SEM images of typical as-cast CMSX-4^®^ microstructures in under 90 s per image. Such accuracy is achieved due to a sequence of specially selected image pre-processing methods. Coupled with statistical analysis the model has the potential to gather large quantities of structural data accurately and rapidly, allowing for optimisation and quality control of industrial processes to improve mechanical and creep performance of materials.

## 1. Introduction

The dendritic structure is one of the most complex forms of crystallisation in nature and technology. In directionally solidified alloys, primary dendrite arm spacing (PDAS) controls the maximum length scale for segregation [1,2,3], which determines the propensity for defect formation [3,4], the solutioning heat treatment time [5], and mechanical properties [6]. It has been found that for the same processing conditions, a significant range of PDAS exists [7,8,9] with the maximum being up to four times the minimum. A large PDAS is associated with a greater non-uniformity of composition i.e., microsegregation. This results in the formation of low melting point secondary phase eutectics, as well as incoherent precipitates and pores in the interdendritic region. It is these secondary phases and defects which determine the properties of the as-cast material and its high temperature performance. Solution heat treatments are applied to remove the eutectics and homogenise the microstructure, where solutioning time is proportional to the square of the PDAS [10]. It follows, that accurate determination of PDAS is crucial as it is directly associated with the level of microsegregation, defect formation, and material properties.

Classical methods to determine the average [11,12,13] and local [14,15,16,17,18,19] PDAS, all require laborious manual determination of the dendrite cores. The manual counting process takes approximately 20 min to 1.5 h depending on the size of the sample surface and number of dendrites. It is performed on every new batch of samples with a process or chemistry change, during furnace calibration, and on every newly developed cast component. This overwhelming amount of data, and thus time, creates a bottleneck at the core identification and PDAS calculation step which must be addressed.

**Figure 1:**
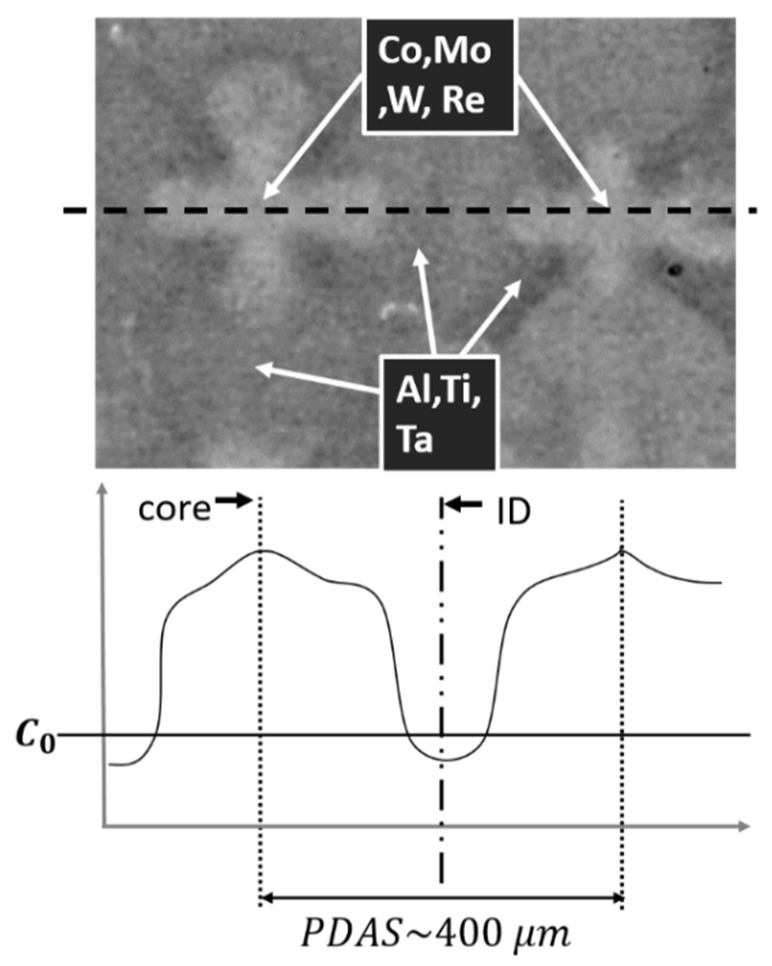
A micrograph of two dendrites from a directionally solidified CMSX-4^®^ alloy, showing elemental microsegregation with heavy elements such as Co, Mo, W, and Re across the dendrite core and lighter elements, Al, Ti, and Ta, in the interdendritic regions (ID). The heavy and the light elements cause the contrast between the brighter and darker regions, respectively. An approximate primary dendrite arm spacing (PDAS) distance and nominal composition, $C_{0}$, are shown for reference.

Dendritic microstructures are typically imaged by backscattered electrons during scanning electron microscopy (SEM). Microsegregation induced compositional variation in dendritic and interdendritic regions provides clearly observable contrast. As shown in Figure 1, dendritic cores appear brighter due to the greater elastic scattering of incident primary electrons resulting from the higher concentration of heavier elements.

As seen in Figure 1, the dendrite core has a unique shape and clearly defined features that resembles a symbol of a cross; it is easily recognisable for humans, but less simple for detection by computer. An algorithm is required that can distinguish between the core and other redundant features; in a sense, create a tool for robot-vision.

A previous attempt to create such a tool was performed by Tschopp et al. [14,15], who developed an algorithm to calculate quantitative four-fold symmetry parameters for the dendrite structures in optical images, with a reported accuracy in core detection of 87.7%. However, the algorithm requires manual intervention to remove mounting material, eutectics, and defects. Due to the convoluted algorithm route, pre- and post-processing, the method requires extensive computational times. The accuracy and automation in their method has reached its limits, therefore a new extraction technique requires development.

**Figure 2:**
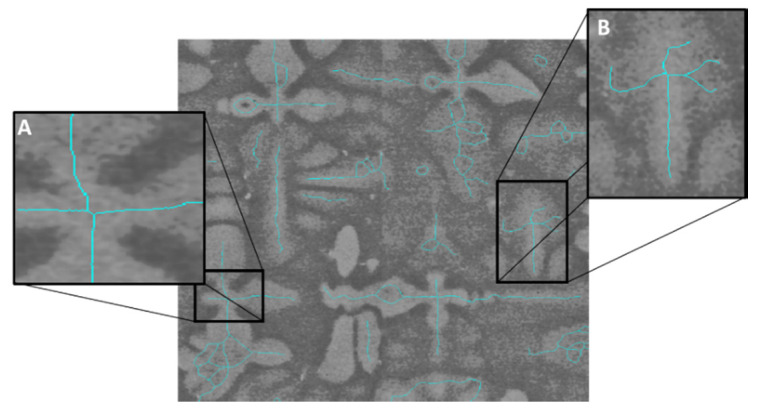
Micrograph with skeletonised dendrites, (**A**) a zoomed-in section of a skewed dendrite core cross and (**B**) a secondary arm representing a dendrite core cross. Image not to scale.

Miller et al. [20] applied a skeletonisation technique which erodes a binary SEM image, whereby dendrites are morphed into a pixel thin line, as seen in Figure 2. This dendritic centre detection algorithm relies on identifying binary crosses and performing statistical comparison between lengths of branches. The accuracy of such an algorithm is poor because intersections at the dendrite core can diverge massively from an ideal binary cross and a large variety of branch lengths and random features are possible, see Figure 2.

The objective of this work herein is to develop a novel, efficient and accurate pattern recognition algorithm that can automatically detect dendritic cores for an entire bulk microstructure: DenMap.

The new method is tested against SEM images of CMSX-4^®^ (Cannon Muskegon Corporation, Muskegon, MI, USA) single crystal nickel-base superalloy used in aero-engine applications. The eventual aim is to establish an industrially standardised digital tool combined with methods for characterisation optimisation with the purpose of gathering of large data sets for machine learning.

## 2. DenMap—Algorithm for Auto-Detection of Dendrite Cores

### 2.1. Normalised Cross-Correlation (NCC) Algorithm

Currently, there are a number of popular pattern recognition approaches in computer vision literature, such as active contour models, convolutional neural networks (CNN) [21] and normalised cross-correlation (NCC) [22], and an even bigger number of variations and improvements of those. Certain algorithms provide definite advantages for high resolution colour images, however, the SEM images that are analysed in the current work have high intrinsic fuzziness, noise, and highly random features.

Contour methods [23,24] coupled with curvature calculations were first attempted in order to identify and map dendrite cores. However, inherent randomness, meant multiple other objects are also detected, such as, separated secondary arms and numerous globular fragments, which all led to the creation of large interconnected structures with one contour containing multiple centres inside. Furthermore, a standard second order derivative curvature, Gaussian curvature as well as a spline fitting method gave highly fluctuating data with no recognisable pattern. It is concluded that those methods, classed as feature detection algorithms, could be used for pre- and post-processing but are problematic for dendrite core detection.

**Figure 3:**
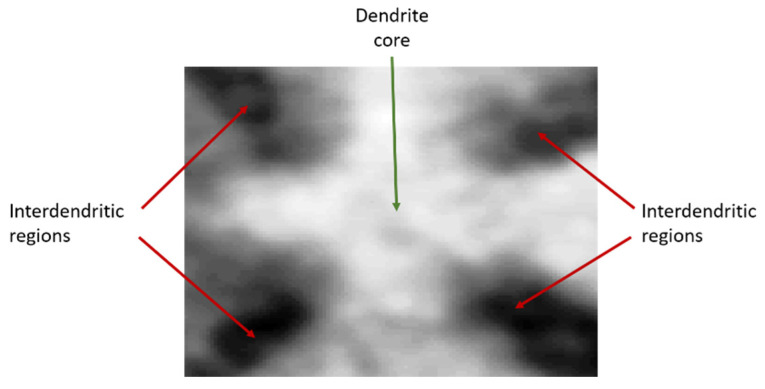
An example of a template for correlation, illustrating clearly a distinguishable dendritic core from surrounding interdendritic regions. As cores are forming in a near steady-state condition they maintain the same shape across an array. Therefore, any dendrite from within the array can be used as a template for the normalised cross-correlation (NCC).

Auto detection is achieved via NCC algorithm, implemented in MATLAB R2018a (The Mathworks, Inc., Natick, MA, USA). It provides a measure of similarity between two data sets or images. The search for the correlation co-efficient is achieved by applying a systematic row-scan. A template (Figure 3), that contains some distinct area with measurable quantities such as brightness and phase regions, is scanned over an image of interest (IOI). The NCC method is less sensitive to changes in brightness between the two input images due to the incorporated normalisation [25]. Unlike feature-based methods [23,24], NCC is an area-based algorithm that matches measurable image quantities such as brightness, absolute gradient, and phase [26,27]. This algorithm has wide range of applications in pattern recognition, including face recognition, industrial inspections, video encoding, MRI imaging, database classification, and mosaicking [25,27].

In the implementation of the NCC, the image is normalised to a unit length, yielding a cosine-like correlation co-efficient (Equation (1)) [22]:

(1) $$\gamma \left(u,v\right)=\frac{\Sigma_{x,y}\;\left[f\left(x,y\right)-{\bar{f}}_{u.v}\right]\left[t\left(x-u,y-v\right)-\bar{t}\right]}{\sqrt{\Sigma_{x,y}{\left[f\left(x,y\right)-{\bar{f}}_{u,v}\right]}^{2}{\;\Sigma }_{x,y}{\left[t\left(x-u,y-v\right)-\bar{t}\right]}^{2}}},$$

where: f is the image, $\bar{t}$ is the mean of the template, and $\bar{f_{u,v}}$ is the mean of f(x,y) in the region under the template. As described by the equation, for each pixel of the IOI, an area around that pixel, with the same size of the template, is compared to the template area and based on that comparison a correlation value is calculated for that pixel. Therefore, to achieve high accuracy the NCC algorithm requires a clear consistent template with a matching contrast, scale, and rotation [28,29]. Scale and rotation are obtained during the image acquisition stage, either manually or directly from the instrument, and are implemented as an input in DenMap at the beginning stage by the operator. The main focus of the work herein is to develop an algorithm based on NCC for the specific application of improving dendritic structure auto detection robustness, speed, and accuracy.

### 2.2. Filtering—Improving Accuracy

Image assembly can cause a number of horizontal and vertical stripes of high and low intensity. This leads to reduced accuracy of detection, especially, if these stripes are obscuring one or more recognisable regions relevant to the template. Furthermore, the contrast variation between stitched parts may lead to a reduced effectiveness of the NCC algorithm. In order for the NCC to work effectively, a consistent similarity between the background and the core is required. However, micrographs may possess a very low signal-to-noise ratio which decreases definition causing inconsistent similarity. In order to develop a software tool that is robust and works for images obtained with various settings, consistent image pre-processing must be applied.

To perform the filtering task, first, a fast Fourier transform band pass filter (FFTBPF) is applied, available in Fiji [30]. The Fourier transform produces an image in the frequency domain where features are suppressed or enhanced based on user-defined parameters. The high cut-off frequency, $f_{h}$, has the most pronounced effect, where increasing the value even by a small amount leads to higher blur and removed definition. In our application some blur is beneficial for detection, however too much removed important features. In order to identify the high cut-off for all image resolutions automatically, the relationship $f_{h}=0.1\times {\left(resolution\right)}^{0.25}$ is applied. For the test images in this work, a high cut-off, $f_{h}$ = 8, equalises the contrast variance, whereas a low cut-off, $f_{l}$ = 150, removes noise. A filtered real-space image is obtained by carrying out an inverse Fourier transform. Further effects of the filter include removal of repetitive horizontal or vertical stripes and noise suppression.

**Figure 4:**
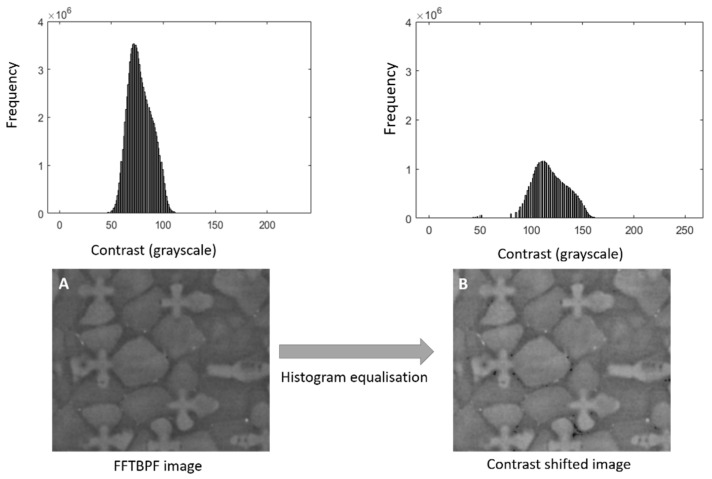
A diagram displaying the changes in an image after applying a histogram equalisation method: (**A**) Fourier transformed image contrast shifted to (**B**) a new reference position; the corresponding changes in the image are shown on a selected image section.

As shown in Figure 4A, the FFTBPF-processed IOI image has equalised the contrast but has also blurred the image and reduced its definition due to the applied Gaussian filter in the frequency domain. This is particularly true for poor quality images. In order to improve the definition in such images, a histogram equalisation filter between the IOI and a reference image is applied. The reference is a small image with a mid-range contrast (90–160) and high brightness. The filter transforms the FFTBPF processed IOI image to match the target histogram, as seen in Figure 4B. As a result, it sharpens all features, helps with identifying noise and black spots and improves the robustness of the NCC algorithm.

**Figure 5:**
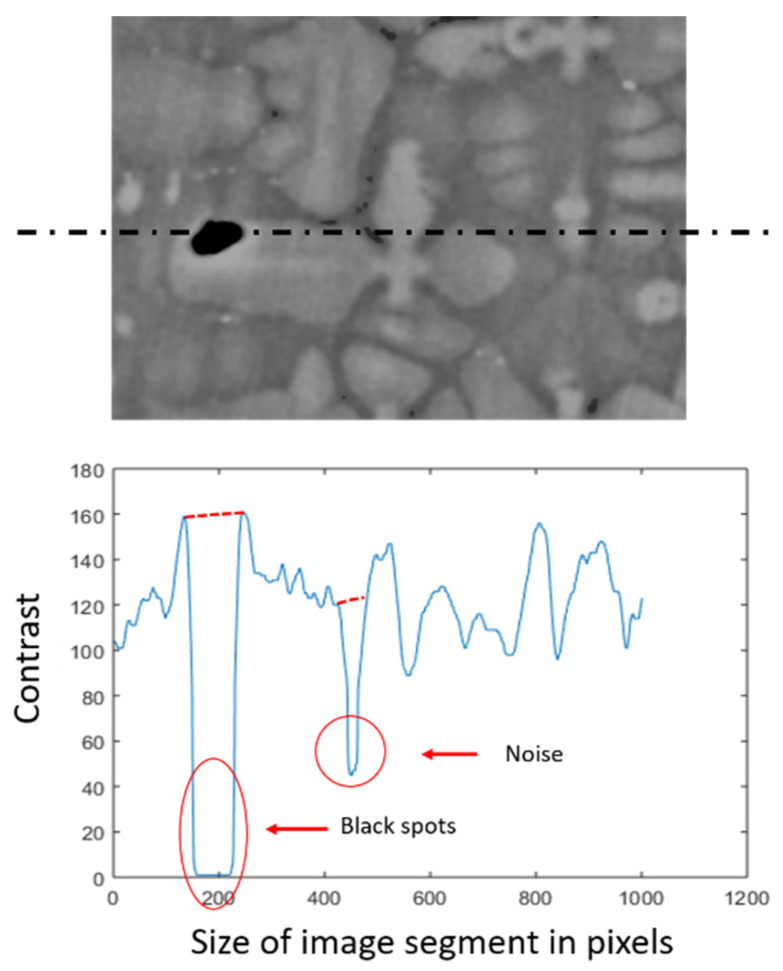
A contrast diagram across a line segment of an image section illustrating black spots and noise and the result of the smearing algorithm applied to those two features (in red).

During solidification the dendritic structures experience large residual stresses resulting in porosity formation. Regardless of the processing conditions or alloy composition, there is always some level of porosity distribution, observed as black spots within the micrographs. If positioned near a dendrite core, these lead to disrupted core detection. As shown in Figure 5, black spots are identified by filtering bellow a threshold value to create a binary mask. A smearing algorithm is then applied to the binary mask, which performs inward linear interpolation from the local neighbourhood values levelling the intensity profile.

### 2.3. Thresholding for Sequential Similarity Detection (SSDA)

**Figure 6:**
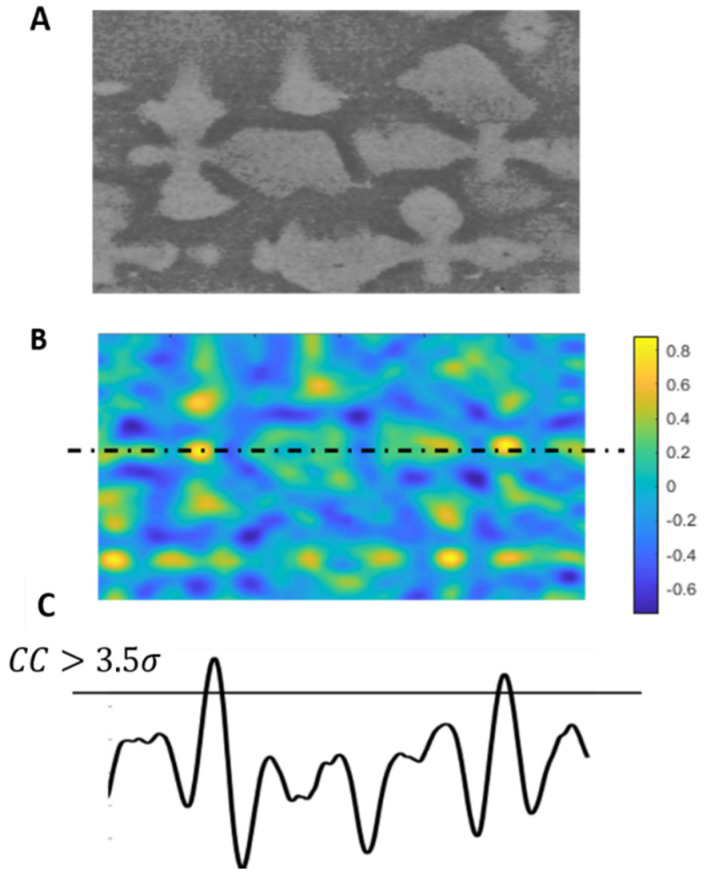
(**A**) Original segment of an image of interest (IOI) image, (**B**) NCC output pattern for a window of size 400 by 500 pixels. (**C**) A representation of the correlation function across the section line, where the correlation coefficients higher than 3.5*σ* identifies only the dendrite cores. Neighbouring peaks correspond to secondary dendrite arms but they are under the thresholding value and not identified.

Once all filtersare applied, both the template and the IOI are input into the NCC algorithm, which follows the general procedure outlined by Lewis et al. [22]. As shown in Figure 6B the result is a map of correlation co-efficient (CC map) spanning between 1 and −1, with peaks indicating high similarity and troughs low similarity, respectively.

The maximum value of the NCC output would identify an exact match, however, a reduced precision of the correlation coefficient would identify similar regions to the template. This method is called the sequential similarity detection algorithm [31,32]. In order to perform SSDA, a threshold value must be applied to the CC map which filters only the highest correlation peaks. It is important to note that secondary arms could also have high similarity with the template and form peaks, as shown in Figure 6C. The following relationship based on a Gaussian distribution of the correlation data is used to calculate the threshold value:

(2) $$\tau_{h}\left(CC\right)=\mu \left(CC\right)+3.5\sigma \left(CC\right),$$

where $\tau_{h}$, is the threshold, μ is the mean, σ is the standard deviation, and CC is the correlation co-efficient. This threshold filters the highest correlation co-efficient that corresponds to a match. The value of 3.5 is found to give the most consistent results with minimum secondary arms captured and most primary cores identified. This value is constant as long as all image processing steps are followed, as tested for 11 SEM images of CMSX-4^®^. The threshold relationship is expected to stay the same for other alloys due to the corresponding change in the template, even though dendrite core dimensions vary with solidification conditions and chemistry.

**Figure 7:**
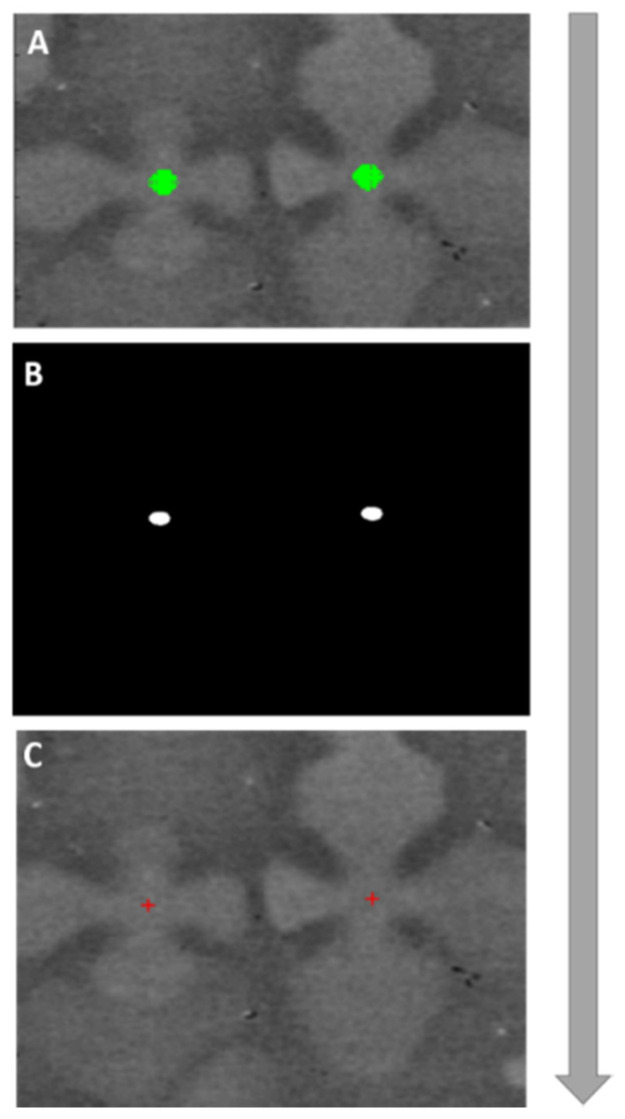
(**A**) scatter of pixel positions of a high correlation region plotted over a segment of the IOI. (**B**) all positions of the correlation peaks mapped into a binary image for the same segment, (**C**) calculated dendrite core positions using a centroid algorithm. Not to scale.

The identified primary peaks have a certain width that spans across several pixels, green detection centres Figure 7A. In 2D, the size of the circular region depends on the goodness of correlation. In order to find the exact coordinates of the dendrite cores, all these regions are indexed and the centroids are calculated. These centroids represent the exact x and y coordinates in pixels of the dendrite cores.

**Figure 8:**
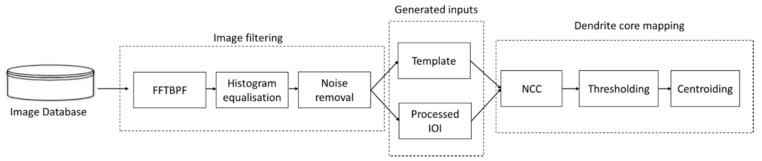
Flow chart for the image processing and detection route of DenMap.

To illustrate the imaging processing route, a schematic is shown in Figure 8. Images from the database are input into a filtering stage which ensures that the template has a consistent contrast with the IOI, improving its accuracy. The processed IOI and template are then generated and analysed by the NCC and the post-processing algorithms. Assuming that scaling and rotation are known, a priori, the characterisation process is automatic.

## 3. Testing and DenMap Performance

**Figure 9:**
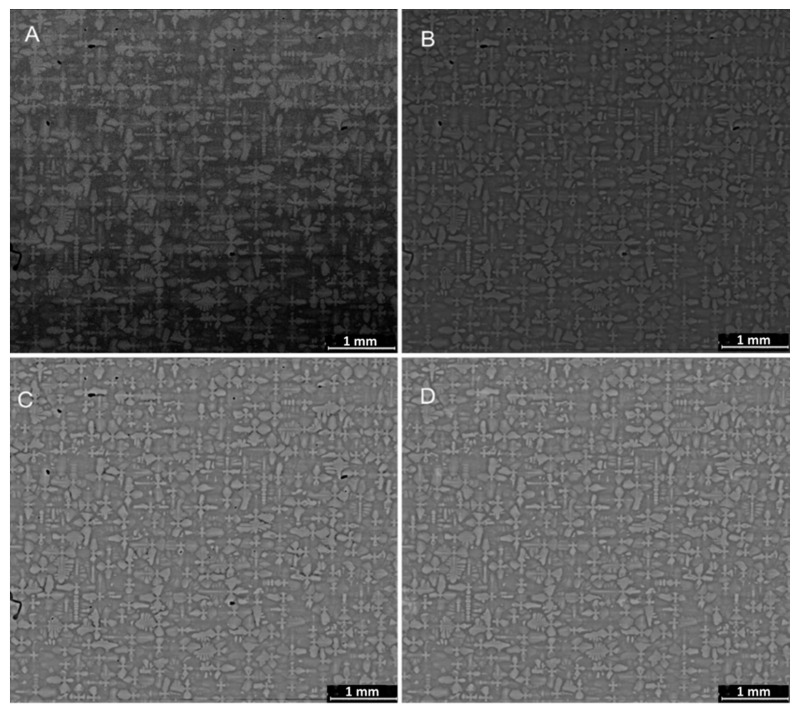
Images at each image processing step: (**A**) the input SEM image (CMSX-4^®^) sectioned perpendicular to the long axes of the sample aligned with heat flow during solidification, (**B**) FFTBPF processed image, (**C**) image after histogram equalisation, and (**D**) image after black spots and noise removal.

In order to test the algorithm and display its performance, a particularly noisy contrast varying image is selected, as seen in Figure 9A. Figure 9B–D then demonstrates the results from following the processing steps of the algorithm, outlined in Figure 8. Image D has most of the noise removed, no black spots and is contrast invariant, which is the precisely desired condition for analysis with NCC.

**Figure 10:**
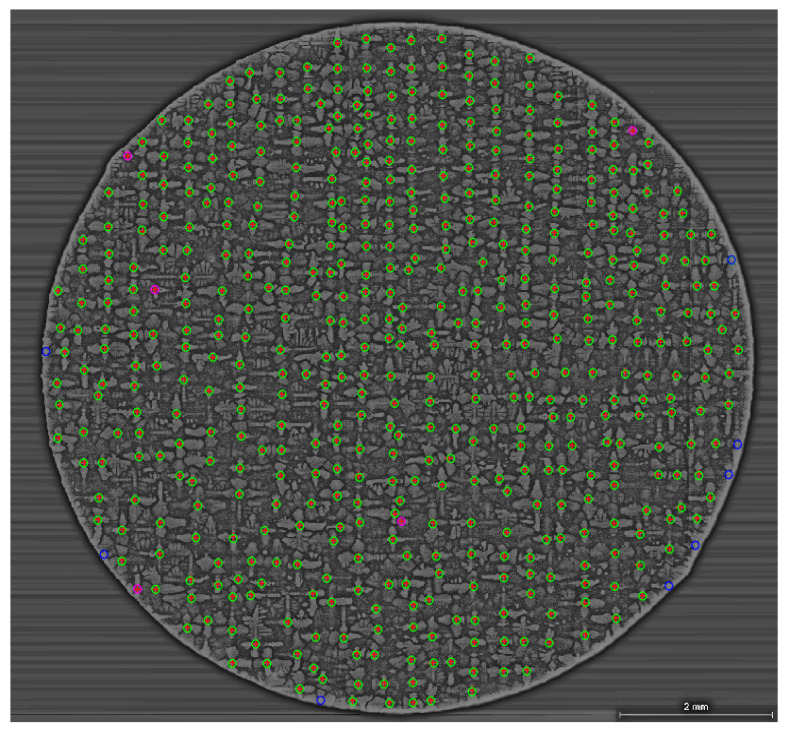
Final processed and mapped image. Dendrite cores are identified with red crosses. Green circles refer to manually selected cores, blue missed out cores, and purple circles detected cores that were omitted from the manual selection. The dendrites highlighted by the purple circles are false positives that have similar shape and intensity to a dendrite core but smaller size and are tertiary or secondary arms in nature.

The algorithm works with high precision when employed for a whole sample bar scan, 9 mm in diameter as seen in Figure 10. No manual intervention is required to achieve this result, even the mount material is filtered automatically by the FFTBPF. The image in Figure 10 has a size of 8947 by 9271 pixels or 9.5 mm by 10 mm. The total processing time from loading the image to recording and plotting the result is 89.41 s, where the FFTBPF took 46.33 s and the NCC algorithm took 16.83 s; run on a PC with Windows 10 (Intel^®^ Core™ i5-8500 CPU @ 3.00 GHz, 16 GB RAM). The accuracy of detection is 98.4% (506 cores detected). The red crosses are the dendritic cores identified by the NCC algorithm; for comparison, the green circles indicate manually counted cores and blue circles highlight cores undetected by NCC. As observed, undetected cores are positioned at the edge of the sample bar. Edges of the image have high brightness and conceal parts of the dendrite cores. The purple circles indicate cores that are not manually identified by the operator but are mapped by the algorithm i.e. false positives. These cores have very high brightness, similar to regular core, but smaller in shape. The suggested possibility for their origin are newly formed tertiary arms. In this analysed sample section, not a single secondary arm is detected as a false positive which points to the confidence of the threshold value for correlation.

**Figure 11:**
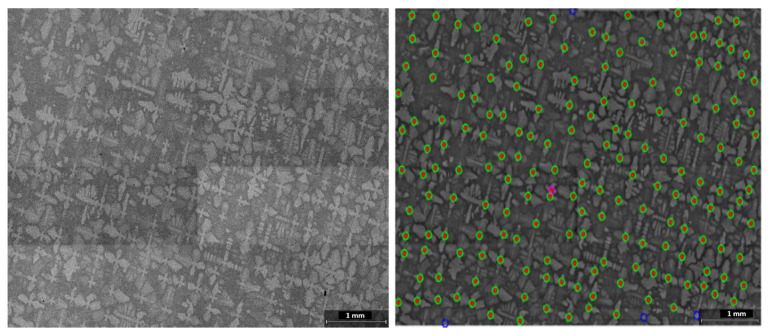
Mapping of a CMSX-4^®^ SEM image with dendrites at an angle. Dendrite cores identified with red crosses. Green circles refer to manually selected cores, blue circles missed out cores, and purple circles NCC detected cores undetected in the manual selection. The dendrites highlighted by the purple circles are false positives that have similar shape and intensity to a dendrite core but smaller size and are tertiary or secondary arms in nature.

To test the robustness of the algorithm, a case where the dendrites are rotated by 15° is investigated, as shown in Figure 11. For the purpose of this test, the template is given the expected angle of the dendrites in the image. Mapping the cores of the rotated dendrites is successful, with most dendrite cores detected. The accuracy of detection in this test case is 97.8%, where red crosses indicate detected dendrite cores, green circles manually selected cores, and blue missed out cores. As seen from the image, two of the undetected cores are located near the scale bar and another two are at the image edge. However, as a result of the noise removal stage, even cores that have black spots in the core region are detected.

In order to further test the robustness of the algorithm, DenMap is applied to multiple image sets. All data in Table 1 is collected using the implemented automatic thresholding. The total accuracy is 98.4%, where the consistency of the results indicates that the algorithm is suitable for industrial applications. In order for the algorithm to perform with a similar to the stated accuracy, it is recommended to use images with dendrite core sizes of at least eight by eight pixels. The false positives arise from tertiary or secondary arms that have similar shape and contrast to a core. Furthermore, the algorithm is independent on the slight local misorientations of the dendrites (−5° to 5° from the Template) as tested for all image cases. The spatial tolerance of the detected dendrites is within three pixels or 4 µm in the investigated examples causing an insignificant ±1% error. Overall, DenMap worked with high speed and accuracy for all SEM CMSX-4^®^ test images.

## 4. Conclusions

DenMap is an automatic pattern recognition tool developed for optimisation of single crystal characterisation. Due to the effective application of smart filtering techniques, an NCC algorithm is successfully implemented to perform dendrite core detection. A micrograph of 9000 by 9000 pixels can be automatically processed in less than 90 s with a dendrite detection accuracy of 98%. DenMap is robust, accurate and consistent and has demonstrated its versatility when applied to nine CMSX-4^®^ SEM micrographs which solidified under various processing conditions. No manual intervention is required for any of the algorithm stages. The pre-processing route successfully handles the mounting material, contrast invariance, and image noise. DenMap has the potential to facilitate accurate and rapid analysis of image databases, to help the optimisation of industrial processes and develop machine-learning algorithms.

## Figures and Tables

**Table 1:** Evaluation of DenMap for a data set of samples of CMSX-4^®^. The first three letter-number combination indicates the sample code and the last letters indicate the batch number.

Sample Code	Image Resolution	Total Number of Cores	Numbers of Detected Cores	False Positives	Missed out Cores	Accuracy (%)
P73L	4772 × 7005	224	221	0	3	98.7
P73R	6550 × 7002	266	268	3	1	98.5
P82L	5592 × 8369	250	250	2	2	98.4
P82ZL	5556 × 8423	247	248	2	1	98.8
P82ZR	6553 × 7033	243	236	0	7	97.1
R13AL	5595 × 5641	252	253	3	2	98.0
R13AR	5620 × 5633	255	257	3	1	98.4
R13ZL	4690 × 7044	209	210	2	1	98.6
R13ZR	5633 × 7031	256	257	2	1	98.8
